# Does rainfall or temperature influence antipredator vigilance in a hibernating mammal?

**DOI:** 10.1093/beheco/araf105

**Published:** 2025-09-20

**Authors:** Karsten Bobb, Katie A Adler, Julien G A Martin, Daniel T Blumstein

**Affiliations:** Rocky Mountain Biological Laboratory, Box 519, Crested Butte, CO 81224, United States; Rocky Mountain Biological Laboratory, Box 519, Crested Butte, CO 81224, United States; Department of Ecology and Evolutionary Biology, University of California, 621 Young Drive South, Los Angeles, CA 90095-1606, United States; Rocky Mountain Biological Laboratory, Box 519, Crested Butte, CO 81224, United States; Department of Biology, University of Ottawa, 30 Marie-Curie, Ottawa, ON, Canada K1N 6N5; Rocky Mountain Biological Laboratory, Box 519, Crested Butte, CO 81224, United States; Department of Ecology and Evolutionary Biology, University of California, 621 Young Drive South, Los Angeles, CA 90095-1606, United States

**Keywords:** antipredator behavior, climate change, thermal plasticity, thermoregulation, yellow-bellied marmots

## Abstract

As the global climate changes, temperatures are rising, snow is melting earlier, and rainfall is becoming more variable, and these climatic changes may create an ecological mismatch. While prior work has shown how animals respond to these changes physiologically and behaviorally, few have specifically investigated antipredator behavior, an essential activity. In many species, there are direct fitness tradeoffs between allocating time and energy to antipredator vigilance and foraging. To discover how these tradeoffs are affected by climate change, we studied how temperature, snowmelt date, and rainfall affected the proportion of time yellow-bellied marmots (*Marmota flaviventer*) allocated to vigilance during bouts of foraging. While snowmelt and temperature did not explain variation in vigilance, rainfall did. Higher rainfall in the week prior to a focal observation was associated with higher vigilance, possibly reflecting more abundant food that affords the luxury of increasing antipredator vigilance while foraging. Such an effect might be consequential at the population level given the importance of foraging and antipredator behaviors for a highly time restrictive hibernating species. Further research is necessary to determine consequences at the population level and whether and how these findings extend to other species.

## Teaser

We found that as total rainfall over a seven-day period increases, yellow-bellied marmots allocate more time to antipredator vigilance. Periods of high rainfall may increase food abundance. As such, individuals may still be able to maximize their food intake prior to hibernation while also prioritizing vigilance towards predators.

## Introduction

Recent reports on climate change present evidence of increasing global temperatures and increased frequency and intensity of natural disasters, such as heatwaves and droughts (IPCC 2023). Alpine and arctic ecosystems have been identified as particularly vulnerable to this warming (IPCC 2023). Many studies have investigated how climate change has impacted animal physiology and behavior, including how individual plasticity may aid organisms in adapting to environmental change. In particular, numerous studies have explored how higher temperatures have impacted animal thermoregulation and activity levels, which then mediate animal behavior (Cooper et al. 2019; Funghi et al. 2019; Mella et al. 2024).

Heat stress is of concern for endotherms because their metabolic processes naturally produce heat, so their energy expenditure is limited by how much heat they can dissipate (heat dissipation theory; Rogers et al. 2021). For instance, koalas (*Phascolarctos cinereus*) lowered their body temperature in the morning of a particularly hot day as if in preparation for the heat (Mella et al. 2024). Cheetahs (*Acinonyx jubatus*) and zebra finches (*Taeniopygia guttata*) adjusted foraging and hunting times, respectively, during heat waves, with cheetahs becoming more crepuscular and zebra finches foraging more in the cooler morning hours when afternoon temperatures were anticipated to be extremely high (Funghi et al. 2019; Hetem et al. 2019). Temperature can alter temporal and energetic tradeoffs, either directly through increased time and energy devoted to thermoregulatory behaviors like seeking shade or escaping to burrows (thermal refugia), panting or licking (evaporative heat loss), and splooting, tree-hugging, or other forms of expelling heat through conduction (Buchholz et al. 2019) or indirectly by altering other processes like metabolic rate (Biro et al. 2010) and cognitive function (Soravia et al. 2023). Typically, more time and energy is allocated to the activity with the greatest fitness consequences (Perrin and Sibly 1993), but for some organisms, competing activities have relatively equal weight in terms of survival and/or reproduction. For example, yellow-bellied marmots (*Marmota flaviventer*) must balance allocating time and energy to foraging to gain enough mass to survive winter hibernation while also allocating time and energy to vigilance to avoid predation and ensure summer survival (Blumstein et al. 2006).

The study of temperature effects on antipredator behavior is especially important because of these tradeoffs and the direct fitness outcomes of antipredator behavior modifications. Changes in antipredator behavior immediately impact organisms’ survival rates by altering their vulnerability to predation. This may have implications for predator–prey dynamics and species survival. However, these impacts are often nuanced because behaviors that reduce the likelihood that an organism is attacked may increase its chances of capture and vice versa (Lind and Cresswell 2005).

Existing research on how temperature affects antipredator behavior has focused extensively on ectotherms, given the direct relationship between temperature and performance for this group. Results vary by species and individuals and often illustrate the presence of individual thermal plasticity (Gomes et al. 2002; Mori and Burghardt 2004; Biro et al. 2010; Briffa et al. 2013). Some ectotherms, like Sousa's snouted tree frogs (*Scinax hiemalis*), guppies (*Poecilia reticulata*), and some species of snakes (such as *Coluber constrictor*, *Natrix maura*, and *Sistrurus catenatus*) are more active, aggressive, and/or have more sensitive antipredator responses at higher temperatures while others, including damselfish (*Pomacentrus chrysurus*) under restricted food availability and other species of snakes (such as *Thamnophis radix*) responded more passively or not at all to predatory stimuli at higher temperatures compared to lower temperatures (Weetman et al. 1998; Gomes et al. 2002; Mori and Burghardt 2004; Lienart et al. 2014).

Only a few studies have investigated temperature effects on antipredator behavior for endotherms and are primarily focused on birds. One such study found that in addition to heat impacting animal behavior through metabolic rate changes, elevated temperatures impair cognition, which can significantly hinder an animal's antipredator behavior (Soravia et al. 2023). Not only does heat stress impact an animal in the present, but it can also have long-term effects. For example, southern pied babblers (*Turdoides bicolor*) that experienced heat stress during rearing were shown to have diminished learning performance and foraging and reproductive success as adults (Soravia et al. 2024). We know comparatively less about mammals' antipredator responses to heat.

Yellow-bellied marmots are hibernating, ground-dwelling, sciurid rodents and are an ideal species to start filling this knowledge gap because they experience a direct tradeoff between allocating time to vigilance (to survive the summer) and foraging (to gain enough mass to survive winter hibernation) (Blumstein et al. 2006). Furthermore, we studied marmots in the upper Colorado River Basin, which has suffered from increasingly severe heatwaves and drought due to decreased snowpack, increased winter melt, and falling stream flows, all of which are compounded by rising air temperatures, making it an appropriate location to study the effects of climate change on antipredator behavior (McCoy et al. 2022; Bolinger et al. 2023). Existing research has shown that marmots avoid being active during extreme temperatures (too hot or too cold), but if forced by energy requirements to forage in unfavorable thermal conditions, they tend to engage in short bouts of foraging with breaks to cool off on rocks in the wind (Melcher et al. 1990). Interestingly, marmots sometimes foraged in extreme temperatures when they had not used all available opportunities to forage in moderate temperatures at another point in the day (Melcher et al. 1990). This could be due to foraging limitations imposed by the time required for digestion, which has been found to restrict foraging behavior in some small homeotherms (Weiner 1992). Marmots have a large cecum that necessitates periods of fermentation digestion between bouts of foraging (Armitage 2014). This added digestion constraint makes marmots particularly interesting to study. Herbivores, including marmots, experience additional thermoregulatory challenges in high temperatures because the detoxification of consumed plant secondary metabolites hinders thermoregulation, making heat stress of increased concern for these animals (Beale et al. 2018).

Much is known about marmot antipredator behavior that permits us to develop focused hypotheses. For instance, several prey-specific factors influence vigilance, including group, environmental, and internal state conditions (Chmura et al. 2016). Socially, mammals foraging in smaller groups (Carey and Moore 1986; Beauchamp et al. 2021), with juveniles present, and on the outside of the group (Di Blanco and Hirsch 2006) are typically more vigilant. Alarm calls by conspecifics, especially juveniles, elicit increased vigilance in fellow marmots (Blumstein and Daniel 2004; Blumstein et al. 2008). Environmentally, marmots are less vigilant on steep slopes (Blumstein et al. 2004) and more vigilant when their view is obstructed (Bednekoff and Blumstein 2009). Animals farther from safety (ie, burrows) are also more vigilant (Carey and Moore 1986; Stankowich and Blumstein 2005; Mateo 2007). Marmots in areas of higher human activity spend more time being vigilant than foraging, but become less sensitive to human approach over time (Uchida and Blumstein 2021). Finally, individual factors, such as personality, with increased boldness leading to less vigilance and greater risk-taking (Dammhahn and Almeling 2012); age, with juveniles being less vigilant, perhaps due to higher energy requirements (Carey and Moore 1986; Bachman 1993; Arenz and Leger 2000; Bednekoff and Blumstein 2009); sex, with males being more responsive to alarm calls (Lea and Blumstein 2011a) and female vigilance being more impacted by social group size (Mady and Blumstein 2017); body condition, with faster, healthier marmots being more responsive to predators (Blumstein et al. 2004; Lea and Blumstein 2011b); stress levels (Mateo 2007); and illness, with infections corresponding to decreased antipredator response (Crane et al. 2011); impact animal vigilance (Chmura et al. 2016). These factors provide insights into how environmental and physiological characteristics may impact vigilance while exposing a need for additional study of climate effects on animal vigilance.

To study the potential impacts of temperature, rainfall, and length of growing season on marmot vigilance, we quantified the proportion of time being vigilant in systematically collected two-minute foraging focal observations (hereafter “focals”). Key measurable variables in our system included temperature when foraging, date of snowmelt, and summer precipitation. We made the following predictions.

First, if marmots forage for shorter intervals of time during extreme temperatures (Melcher et al. 1990), and energy requirements remain constant (or increase), we predict marmots will allocate less time to vigilance as the temperatures rise.

Second, if early snowmelt corresponds to a longer growing season and increased food availability (Van Vuren and Armitage 1991), we anticipate marmots will be more vigilant when snow melts early, as seasonal food intake requirements (to achieve sufficient mass for winter survival by the end of the summer) will be spread out over more days. This may reduce their daily energy requirements and allow for more vigilance while foraging.

Finally, marmot growth rates decrease in drought conditions, particularly for juveniles (Lenihan and Van Vuren 1996; Armitage 2013). This is likely due to an increased resting metabolic rate that marmots experience during droughts (Armitage 2014). If marmots must allocate more time to foraging to meet the higher energy requirements that come with an elevated metabolic rate, we hypothesize that less time will be allocated to vigilance as rainfall decreases.

## Methods

### Study area and marmot ecology

We studied marmots in and around the Rocky Mountain Biological Laboratory (RMBL) in Gothic, Colorado, USA (38°57′N, 106°59′W) from 2002 to 2019 during the marmot active season (April-September). Gothic is located in a subalpine ecosystem (>3,000 masl) in a broadleaf-coniferous mixed forest along an elevational gradient of about 300 m. Snow melts about two weeks later at the higher elevation sites and is often fully melted by early June. There is a monsoon season through July that brings near-daily heavy rains. Terrain is usually grassy, rocky, or both, and hosts a wide variety of wildflower species. Marmot colonies are located on both steep slopes and level terrain.

Yellow-bellied marmots are large, social, semi-fossorial ground squirrels and generalist herbivores that live primarily at high elevation in the Northwestern USA (Feldhamer et al. 2003). Marmots consume a variety of plant species, including *Claytonia lanceolata, Potentilla gracilis*, and *Taraxacum officinale*, with differing diet preferences occurring at different stages of the growing season (Frase and Armitage 1989). Over the growing season, marmot body mass dramatically increases in preparation for hibernation, with adults often around 3 kg at the start of the season and 5 to 6 kg at the end of the season. They are prey to several species across our field site, principally: coyotes (*Canis latrans*), badgers (*Taxidea taxus*), American martens (*Martes americana*), black bears (*Ursus americanus*), red foxes (*Vulpes vulpes*), mountain lions (*Felis concolor*), wolves (*Canis lupus*), and raptors like red-tailed hawks (*Buteo jamaicensis*) and golden eagles (*Aguila chrysaetos*) (Van Vuren 1991; Van Vuren 2001; Armitage 2004).

### Trapping and marking

Marmots at each colony were trapped using Tomahawk live traps baited with horse feed mixed with peanut butter. Traps were placed at all active burrow entrances (if possible) and were checked within 1 to 3 h after being set. During processing, each marmot was tagged with a metal size 3 self-piercing strap tag on each ear and given a mark on their back using Nyanzol fur dye (Armitage 1962). This ensured certain identification of marmots by ear tags when trapped and allowed for identification of marmots by fur mark at a distance (20 to 150 m) during observations.

### Quantifying vigilance while foraging

We observed marmots using spotting scopes from a far distance (20 to 150 m) to avoid behavioral interference. During regular observations (0,700 to 1,100 h and 1,600 to 1,900h), we identified a focal foraging individual and, for two minutes, used continuous recording methods and an established ethogram (Table 1) to score the duration and instance of each behavior. In addition to the marmot's identity, we noted: the time; date; colony; number of other marmots within a 10-m radius; the slope, angle, and substrate (dirt, stones, talus, low vegetation, or high vegetation) where the marmot foraged. Focals were dictated into voice recorders and scored using JWatcher 1.0 (Blumstein and Daniel 2007). Using JWatcher, we calculated the proportions of time in sight that the focal marmot allocated to vigilance (stand look and rear look, Table 1) while engaged in active bouts of foraging. Prior to entering focals, each observer conducted a consistency check and was required to have an intraclass correlation score of at least 0.95 to ensure scoring precision.

**Table 1.** Behaviors scored during a foraging focal (following Chmura et al. 2016).

Behavior	Description
Stand forage (f)	The marmot was on all four legs with its mouth toward the vegetation and head down
Rear forage (g)	The marmot stood on its hind legs and ingested vegetation
Stand look (l)	The marmot was on all four legs with its head up, looking
Rear look (r)	The marmot stood on its hind legs only with its head up, looking
Walk (w)	The marmot walked
Run (n)	The marmot ran
Out of sight (o)	The marmot was out of the view of the observer
Other (t)	The marmot did something other than the actions described above (eg, participating in a social interaction)

### Environmental data collection

Hourly temperatures in the upper East River Valley (where the marmot colonies are located) were collected from the Gothic Research Meadow weather station for each hour that a focal was recorded. The time each focal was taken was rounded to the nearest hour to synchronize the focal time with the time of the closest temperature recording (Gothic Weather 2024). Focals were recorded within 2.5 km of the weather station, so the temperature recordings are somewhat relative rather than absolute.

Marmot colonies vary in size, but average about 2 ha (Armitage 2014). We defined marmot colonies by drawing polygons around extreme locations where marmots were seen. Using the RMBL Spatial Data Platform (https://www.rmbl.org/scientists/resources/spatial-data-platform/), we measured the date for each colony in a given year at which snow was no longer detected via remote sensing at ≤ 3 cm resolution. This allowed us to quantify the start of the growing season for plants and the active season for marmots.

Rainfall across multiple time periods is known to have cumulative effects on vegetation growth (Ding et al. 2020). We calculated the total rainfall across the seven, fifteen, and thirty days prior to the focal (including the day of focal observation) to analyze how recent rainfall impacts foraging tradeoffs. Daily rainfall was collected from a private weather station (maintained by billy barr) adjacent to the Gothic townsite.

### Statistical analyses

Before analysis, we recategorized substrate to combine low vegetation (LV) and dirt (D) since both surfaces are easily maneuverable and afford good visibility, while talus (T) and stones (S) were combined because these substrates are similar in their difficult maneuverability. High vegetation (HV) remained its own category as it provides poor visibility and difficult maneuverability. Following Chmura et al. (2016), only focals lasting 60 s or longer were included in the analysis to exclude observations where marmots were out of sight for the majority of the focal.

We fitted a generalized linear mixed model to explain variation in the proportion of time allocated to vigilance during a bout of foraging using the package “glmmTMB” (Brooks et al. 2017). We used the function “glmmTMB” with “family = ordbeta”. We fitted the following fixed effects: temperature, date of snowmelt, rainfall within the past seven days, valley position (up valley or down valley), day of year, time that focal was collected, number of marmots within 10 m, substrate (high vegetation, low vegetation, stones, and talus), age class (juvenile, <1 yr; yearling, 1 yr; adult, ≥2 years), and sex. We included marmot identity, year, and colony as random effects. All continuous variables were scaled in R (Version 4.4.1; R Core Team 2024) using the “scale” function. Our full data set used for these analyses contained a total of 2,964 observations from 652 individuals over their lifetimes (308 males, 344 females; 197 adults, 401 yearlings, and 259 juveniles) studied across 18 years, which corresponds to 113 hours of focal observations. We also fitted two additional generalized linear mixed models with total rainfall within the past fifteen and thirty days to determine whether rainfall effects differed depending on the amount of time considered (see Supplementary material).

In addition to these main effect analyses, we examined interactions between age class and our fixed effects of interest (snowmelt date, temperature, and rainfall) and between sex and these fixed effects. This allowed us to ask whether the varying energy requirements of different age groups and sexes influenced the relationship between our fixed effects and dependent variable (vigilance). We found that there was no interaction between rainfall and age class or temperature and age class and that there was no significant effect of the other interactions on vigilance, so we dropped all interactions from the final model.

All statistical analyses and modeling were conducted using the R programming environment (Version 4.4.1; R Core Team 2024). We used the packages “sjPlot” (Version 2.8.16; Lüdecke 2024) and “patchwork” (Version 1.2.0; Pedersen 2024) to visualize the data, and checked distributional assumptions for our models using the packages “performance” (Version 0.12.0; Lüdecke et al. 2021) and “DHARMa” (Version 0.4.6; Hartig et al. 2024).

## Results

**Fig. 1.**
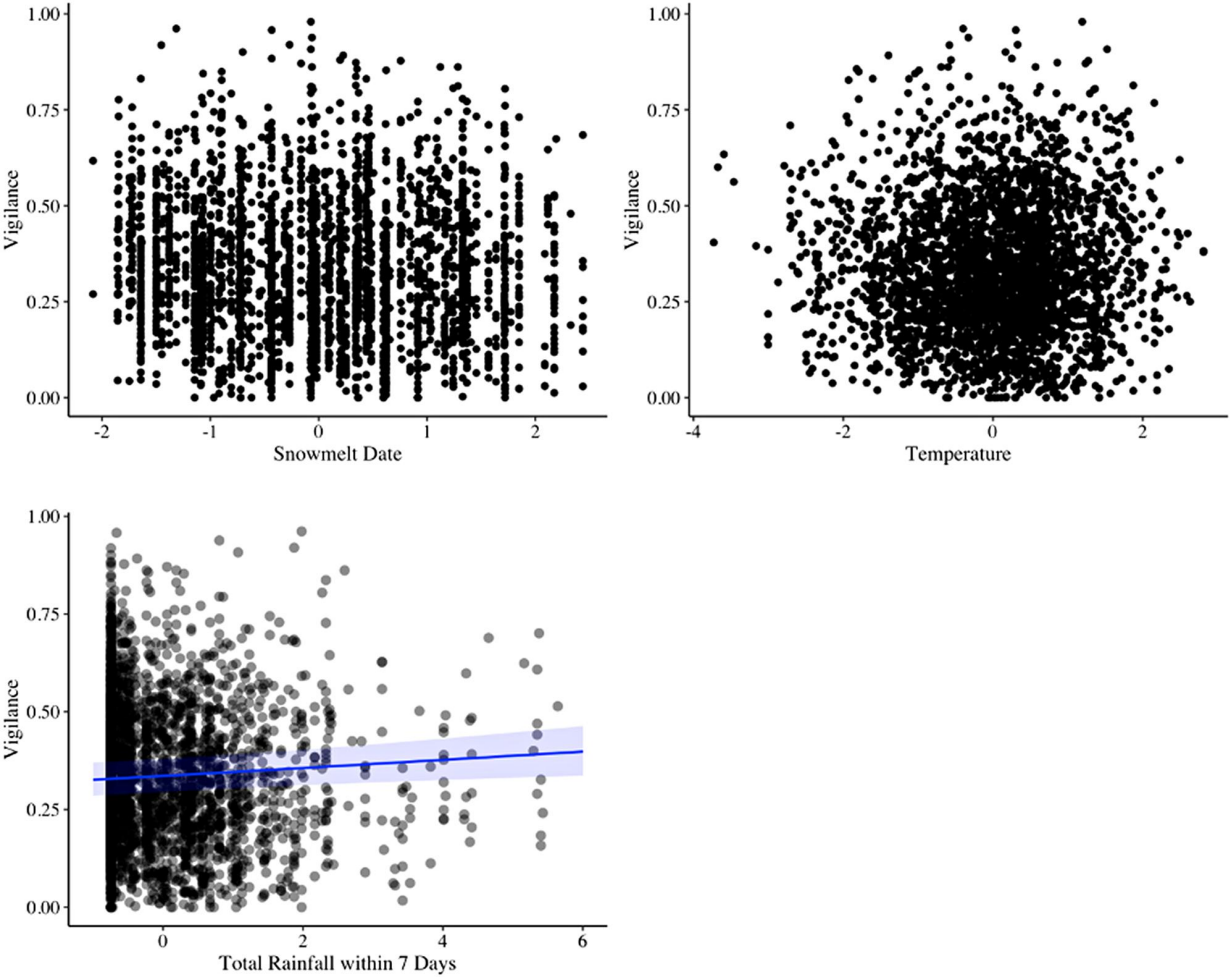
Snowmelt, temperature, and rainfall effects on time marmots allocate to vigilance while foraging. All variables were scaled (root mean squared). The points represent individual observations, and the line and shaded region represents a line of best fit and margins of error with a 95% confidence interval. Rainfall in the preceding seven days of a focal observation explained significant variation in time allocated to vigilance.

Snowmelt date ranged from 17 April to 19 June, and temperature ranged from −0.1°C to 24.2 °C. Neither snowmelt date nor temperature had a significant effect on yellow-bellied marmot vigilance while foraging (Table 2; Fig. 1). Total rainfall within the previous seven days ranged from 0 mm to 60.7 mm. Rainfall had a significant positive impact on marmot vigilance (estimate = 0.044, *P* = 0.006; Table 2; Fig. 1). Some of the fixed effects included in the models had significant impacts on marmot vigilance, which was mostly consistent with prior studies (see Discussion). Foraging in low vegetation/dirt substrates (compared to high vegetation), had a significant positive effect on marmot vigilance, while valley position, the number of individuals within 10 m, and juvenile/yearling age classes (compared to adults) had significant negative impacts (Table 2). All variance inflation factors were less than 2. We also found similar significant effects of rainfall from fifteen and thirty days prior on vigilance (Tables S1 and S2).

**Table 2.** Results of the generalized linear mixed model.

Fixed effects	Variable	Estimate	SE	*P*-value
	Snowmelt	−0.007	0.038	0.865
	Temperature	0.007	0.017	0.688
	Rainfall	0.045	0.016	**0.006**
	Valley position (up)	−0.404	0.122	**<0.001**
	Day of year	0.026	0.023	0.241
	Time of focal	−0.003	0.015	0.853
	Marmots within 10 m	−0.087	0.015	**<0.001**
	Substrate (low vegetation/dirt)	0.151	0.036	**<0.001**
	Substrate (stones/talus)	0.186	0.066	**0.005**
	Age class (juvenile)	−0.478	0.059	**<0.001**
	Age class (yearling)	−0.074	0.036	**0.038**
	Sex (male)	<−0.001	0.036	0.982

Random effects		Variance	SD
	Marmot ID	0.039	0.196
	Year	0.022	0.177
	Colony	0.031	0.150

Snowmelt date, temperature, and rainfall effects on time allocated to vigilance while foraging by marmots. For each fixed effect, an estimate, standard error, and *P*-value are reported. For each random effect, variance and standard deviation is reported. The reference categories are as follows: Valley Position—Down, Substrate—High Vegetation, Age Class—Adult, Sex—Female. *P*-values < 0.05 are bolded.

## Discussion

The total rainfall in the seven days prior to a focal observation explained significant positive variation in time allocated to vigilance. However, snowmelt and temperature did not explain variation in time allocated to vigilance. This suggests that weather has a partial impact on marmot antipredator behavior.

Increased rainfall may increase plant growth within a given area (Fang et al. 2005). Higher plant density may provide more food resources for marmots, allowing them to forage more over the growing season and have more time overall for vigilance. Marmots are also sensitive to weather changes (Ferrari et al. 2022); they may change their behavior to be more wary in extreme weather conditions. Thus, following heavy rains, marmots may generally be more vigilant. Increased antipredator wariness after precipitation events is experienced by redshanks (*Tringa totanus*; Hilton et al. 1999) as well, suggesting that rainfall may have substantial impacts on antipredator behavior across taxa. However, extremely few studies have focused specifically on rainfall's impact on antipredator vigilance. As rainfall events become more variable with a changing climate, it will be important to study this phenomenon further because antipredator vigilance is often assumed to be a consequential activity at both individual and population levels.

Our result is further supported by the supplementary analyses that included rainfall within fifteen and thirty days, which had results consistent with our primary model. In all three models, high precipitation was associated with increased time allocated to vigilance. Thus, precipitation may have important effects on immediate and prolonged plant growth across the growing season, with cascading effects on antipredator vigilance.

An important limitation of this result, however, is that we were unable to measure time allocation to vigilance *during* periods of precipitation. Rainfall may influence vigilance through mechanisms outside the scope of our methodology and analyses, such as by impacting visibility or the ability to detect predators. As such, future studies could attempt to quantify vigilance while foraging while it is actively raining (something that in our experience is relatively rare).

Although snowmelt date did not significantly impact time allocation to vigilance, there is evidence that later snowmelt influences sex-specific emergence (Blumstein 2009), social structure (Philson et al. 2024), and summer mass gain (Maldonado-Chaparro et al. 2015). Changes in marmot behavior and physiology may have direct fitness consequences. Therefore, climatic variables such as snowmelt should still be considered as potentially important predictors in future studies.

Temperature could affect general activity. For instance, marmots may adjust the total amount of time they spend foraging or the times of day that they forage in response to rising temperatures and changing rain patterns rather than altering their behavior while foraging (the latter of which we have studied extensively). However, our observations do not allow us to quantitatively assess variation in marmot foraging activity throughout the day. We do not observe marmots under extremely hot conditions because they are less active or entirely inactive, which may be a limitation of our study and explains the lack of a significant relationship between vigilance and temperature during a foraging bout. This is consistent with the behavior of other small mammals, such as desert woodrats (*Neotomia lepida*) and northern flying squirrels (*Glaucomys sabrinus*), who alter their active times to avoid or reduce exposure to extreme hot and cold temperatures, respectively (Cotton and Parker 2000; Murray and Smith 2012). Pikas (*Ochotona princeps*), which live in similar forest-adjacent talus environments to marmots, took refuge in talus burrows to escape the intense heat of a forest fire, which shows the effectiveness of these burrows in sheltering small endotherms from heat and that, behaviorally, these animals take advantage of these resources to thermoregulate (Varner et al. 2015). Similarly to our rainfall result, temperature may indirectly influence marmot vigilance through other mechanisms that we did not measure, such as changes in plant growth. Further study may be important to understanding potential indirect pathways by which temperature may influence antipredator vigilance.

We have confidence in our overall results because several of the fixed effects we included explained variation in time allocated to vigilance. The directionality of these associations was generally consistent with prior studies and illustrates the energetic and temporal tradeoff between vigilance and foraging. Marmots foraging with more conspecifics were less vigilant, a common finding across marmot vigilance studies (Holmes 1984; Carey and Moore 1986; Chmura et al. 2016) and mammalian vigilance studies at large (Quenette 1990). This is likely explained by either the group vigilance or “more eyes” hypothesis (that on the individual level, prey can afford to be less vigilant when they are surrounded by others who are vigilant) or the individual risk hypothesis (that individual predation risk decreases in larger groups due to dilution or confusion effects; thus, less individual vigilance is warranted) (Roberts 1996). Additionally, marmots are less vigilant at higher elevations. Marmots at our higher elevation sites hibernate for 14 days longer than those at the lower elevation sites (Blumstein et al. 2004b). These individuals must allocate more time to foraging as they have less time to gain mass in preparation for hibernation.

Juvenile and yearling marmots were less vigilant than adults, a finding consistent with previous studies in this system (Lea and Blumstein 2011b; Chmura et al. 2016). Juveniles have particularly higher energy requirements in terms of mass gain since they must grow somatically in addition to gaining fat for the winter (Heissenberger et al. 2020), and so they may prioritize foraging more than vigilance. Yearlings, especially females, may prioritize social foraging as a means of social cohesion to avoid dispersal (Blumstein et al. 2009), which may explain their lower overall vigilance. Future studies should focus on age-sex interaction effects on vigilance to further elucidate this finding.

Marmots were more vigilant when foraging in low vegetation and on rocky surfaces, which suggests that greater exposure puts individuals at greater risk of predation, which may increase their time allocation to vigilance over foraging. Interestingly, this is contrary to prior research showing that marmots typically allocate more time to vigilance in high vegetation (Bednekoff and Blumstein 2009; Chmura et al. 2016). Marmots foraging in low vegetation and on rocky terrain usually have more security in that they can see predators from a further distance and can thus allocate more time to foraging (Blumstein et al. 2004). As compared to our analysis, these prior studies used much smaller sample sizes and different covariates to look at time allocation to vigilance and foraging, which may explain some of the variation in the results. This current study has substantially more power because of the larger sample size (>3,000) and should permit us to confidently assert that the trend we observe is likely to be represented in nature.

In summary, we can infer that yellow-bellied marmots may benefit from seasons with increased rainfall because they are able to be more cautious while foraging. Further research should investigate whether other species experience similar shifts in behavior due to rainfall and more generally study the mechanisms by which rainfall affects mammalian survival by changing antipredator behavior. The insights from these studies will permit us to better understand prey population dynamics across climate conditions.

## Supplementary Material

araf105_Supplementary_Data

## Data Availability

Analyses reported in this article can be reproduced using the data provided by Bobb et al. (2025).
