# Supplementary material for: Does rainfall or temperature influence antipredator vigilance in a hibernating mammal?
Source: Behav Ecol. 2025 Sep 20;36(5):araf105. doi: 10.1093/beheco/araf105 (PMC12510307; doi:10.1093/beheco/araf105)
Supplement: araf105_Supplementary_Data [file araf105_supplementary_data.docx]

**Supplement to: Does rainfall or temperature influence antipredator vigilance in a hibernating mammal?**

**Table S1**. Results of the generalized linear mixed model with total rainfall fifteen days prior to focal observations. Our model included 2911 unique observations across 18 years and 644 individuals. All variance inflation factors were less than 2. Snowmelt date, temperature, and rainfall effects on time allocated to vigilance while foraging by marmots. For each fixed effect, an estimate, standard error, and P-value are reported. For each random effect, variance and standard deviation is reported. The reference categories are as follows: Valley Position – Down, Substrate – High Vegetation, Age Class – Adult, Sex – Female.

|  | **Variable** | **Estimate** | **SE** | **P-value** |
| --- | --- | --- | --- | --- |
| **Fixed effects** |  |  |  |  |
|  | Snowmelt | -0.003 | 0.038 | 0.938 |
|  | Temperature | 0.008 | 0.016 | 0.630 |
|  | Rainfall | 0.049 | 0.020 | **0.015** |
|  | Valley Position (Up) | -0.410 | 0.122 | **<0.001** |
|  | Day of Year | -0.032 | 0.023 | 0.159 |
|  | Time of Focal | -0.002 | 0.015 | 0.900 |
|  | Marmots within 10 m | -0.084 | 0.015 | **<0.001** |
|  | Substrate (Low Vegetation/Dirt) | 0.142 | 0.036 | **<0.001** |
|  | Substrate (Stones/Talus) | 0.186 | 0.067 | **0.005** |
|  | Age Class (Juvenile) | -0.454 | 0.059 | **<0.001** |
|  | Age Class (Yearling) | -0.076 | 0.036 | **0.035** |
|  | Sex (Male) | -0.008 | 0.036 | 0.820 |
|  |  |  |  |  |
| **Random effects** |  | **Variance** | **SD** |  |
|  | Marmot ID | 0.037 | 0.193 |  |
|  | Year | 0.022 | 0.148 |  |
|  | Colony | 0.031 | 0.176 |  |

**Table S2**. Results of the generalized linear mixed model with total rainfall thirty days prior to focal observations. Our model included 2811 unique observations across 18 years and 631 individuals. All variance inflation factors were less than 3. Snowmelt date, temperature, and rainfall effects on time allocated to vigilance while foraging by marmots. For each fixed effect, an estimate, standard error, and P-value are reported. For each random effect, variance and standard deviation is reported. The reference categories are as follows: Valley Position – Down, Substrate – High Vegetation, Age Class – Adult, Sex – Female.

|  | **Variable** | **Estimate** | **SE** | **P-value** |
| --- | --- | --- | --- | --- |
| **Fixed effects** |  |  |  |  |
|  | Snowmelt | -0.008 | 0.097 | 0.832 |
|  | Temperature | 0.015 | 0.017 | 0.364 |
|  | Rainfall | 0.065 | 0.025 | **0.010** |
|  | Valley Position (Up) | -0.429 | 0.122 | **<0.001** |
|  | Day of Year | -0.052 | 0.024 | **0.028** |
|  | Time of Focal | -0.003 | 0.016 | 0.838 |
|  | Marmots within 10 m | -0.083 | 0.015 | **<0.001** |
|  | Substrate (Low Vegetation/Dirt) | 0.141 | 0.036 | **<0.001** |
|  | Substrate (Stones/Talus) | 0.201 | 0.067 | **0.003** |
|  | Age Class (Juvenile) | -0.432 | 0.060 | **<0.001** |
|  | Age Class (Yearling) | -0.069 | 0.037 | 0.063 |
|  | Sex (Male) | -0.018 | 0.037 | 0.630 |
|  |  |  |  |  |
| **Random effects** |  | **Variance** | **SD** |  |
|  | Marmot ID | 0.037 | 0.194 |  |
|  | Year | 0.022 | 0.149 |  |
|  | Colony | 0.031 | 0.175 |  |
